# Analysis of Thermal Characteristics of Potato and Hop Pollen for Their Cryopreservation and Cross-Breeding

**DOI:** 10.3390/plants13111578

**Published:** 2024-06-06

**Authors:** Milos Faltus, Jaroslava Domkářová, Petr Svoboda, Vendulka Horáčková, Vladimír Nesvadba, Vladislav Klička, Jiří Ptáček, Alois Bilavcik, Jiri Zamecnik

**Affiliations:** 1 Crop Research Institute, Drnovská 507, CZ161 06 Prague, Czech Republic; bilavcik@vurv.cz (A.B.); zamecnik@vurv.cz (J.Z.); 2 Potato Research Institute, Dobrovského 2366, CZ580 01 Havlickuv Brod, Czech Republic; domkarova@vubhb.cz (J.D.); horackova@vubhb.cz (V.H.); ptacek@vubhb.cz (J.P.); 3 Hop Research Institute, Kadaňská 2525, CZ438 01 Zatec, Czech Republic; svoboda@chizatec.cz (P.S.); nesbadba@chizatec.cz (V.N.); 4 VESA Velhartice, Velhartice, CZ341 42 Kolinec, Czech Republic; klicka@vesa-velhartice.cz

**Keywords:** breeding, cryoconservation, DSC, glass transition, *Humulus lupulus*, pollination, *Solanum tuberosum*, viability, water content

## Abstract

This study investigated the thermal properties of potato and hop pollen for cryopreservation and subsequent cross-breeding. Phase transitions and frozen water content in selected pollen samples were measured using a differential scanning calorimeter (DSC). Unlike hop pollen, potato pollen showed high variability in thermal properties and water content. Three specific types of pollen samples based on their thermal characteristics and water content were distinguished by DSC in potato: (1) ‘glassy’, with a water content lower than 0.21 g water per g dry matter; (2) ‘transient’, with a water content between 0.27 and 0.34 g of water per g of dry matter; (3) ‘frozen’, with a water content higher than 0.34 g of water per g of dry matter. Only the ‘glassy’ pollen samples with a low water content showed suitable properties for its long-term storage using cryopreservation in potato and hops. Cryopreservation of pollen did not significantly reduce its viability, and cryopreserved pollen was successfully used to produce both potato and hop hybrids. The results indicate that cryopreservation is a feasible technique for the preservation and utilization of pollen of these crops in the breeding process.

## 1. Introduction

Cryopreservation is a method of preserving plant genetic resources by storing them at ultra-low temperatures, usually in liquid nitrogen (−196 °C) [1]. This method is mainly applied to plant species that are propagated vegetatively (such as potato and hop) or produce recalcitrant seeds that cannot be stored by conventional methods (such as coffee and cocoa) [2]. Cryopreservation allows the long-term conservation of plant genetic diversity, which is essential for food security, crop improvement and environmental protection [3].

The key principle of cryopreservation is to prevent the formation of ice crystals in the plant cells, which can cause irreversible damage to the cell membranes and organelles [4,5]. To achieve this, the plant material must be dehydrated to a large extent so that the remaining water turns into a glassy rather than crystalline state at low temperatures [6,7]. There are different approaches to achieve this, such as slow controlled freezing, which gradually lowers the temperature and removes the water by sublimation [8], or vitrification, which rapidly exposes the plant material to ultra-low temperature after prior dehydration with a highly concentrated solution of cryoprotectants, which replace the water and prevent ice formation [9,10].

The optimal conditions for cryopreservation depend on the type and physiological state of the plant material, as well as the cryopreservation protocol used. One of the techniques that can be used to measure these conditions is differential scanning calorimetry (DSC) [11,12], which is a thermal analysis method that records the heat flow associated with the physical and chemical changes of a sample as a function of temperature [13]. By using DSC, it is possible to determine the glass transition temperature, which is the temperature at which the sample changes from a glassy to a rubbery state, the freezing or melting temperature, which is the temperature at which ice forms or melts in the sample, and the frozen water content, which is the amount of water that forms ice crystals [14]. The DSC method can be used to optimize the preparation of plant material before its cryopreservation, such as the degree and method of dehydration, the type and concentration of cryoprotectants, and the cooling and warming rates [12,15,16].

One possible application of cryopreservation is the preservation of pollen, which is the male gametophyte of flowering plants. Pollen are haploid cells that carry the genetic information of the paternal parent and are responsible for the fertilization of the female gametophyte, the ovule. Pollen conservation is important for plant breeding and hybridization because it allows the storage and transport of pollen from different sources and locations and overcoming limitations that prevent crossing of plants with different flowering times, self-pollination, or current lack of pollen availability [17,18,19].

Hybridization of potatoes and hops is an example of plant breeding that can benefit from pollen preservation. Potatoes and hops are both vegetatively propagated crops that have a high economic and cultural value. Potatoes are one of the most important staple food crops in the world [20], and hops are the main ingredient for beer production [21]. Hybridization of potatoes and hops aims to improve the agronomic and quality traits of these crops, such as yield, disease resistance, tuber size and shape in potato, and aroma and bitterness in hop. The short longevity and rapid degradation of potato pollen, even when stored in a refrigerator, limits the possibility of crossing genotypes that flower at different times. In addition, cytoplasmic male sterility, commonly found in potato, can be a serious problem and significantly disrupts the breeding process [22]. The dioecious and perennial nature of hops and the difficulty of estimating the influence of the male genotype on cone value and production requires the maintenance of a large number of male genotypes in the field for cross-breeding purposes [23,24,25].

The cryopreservation of potato and hop pollen is not a common practice, and there is limited information on the use of this method for these crops [26,27]. The only source of information on the cryopreservation of potato and hop pollen is the United States Department of Agriculture (USDA), which has a germplasm repository for these crops and has performed some DSC studies of pollen for cryopreservation, and for other plant species [28,29,30,31,32]. On the other hand, there are quite a lot of papers involved in pollen cryopreservation, carried out on more than 170 plant species [33], including tropical species [34], exceptional plants [35] and medicinal and ornamental plant species [36,37], and vegetable crops [25]. Cryopreservation can be a suitable tool for preserving the genetic resources of plants, and also for increasing the efficiency of the breeding program [17]. One of the decisive factors for the longevity of pollen after cryopreservation is the water content in pollen samples [38], which must be reduced, especially its freezable part [17], most often through desiccation [27,39]. Dehydration of pollen can often be limited by the pollen’s sensitivity to dehydration [32] and therefore the water content and its status are among the important parameters to be monitored during cryopreservation [27].

The potato and hop breeders in the Czech Republic have expressed a need to preserve the pollen of these crops using cryopreservation for their use in breeding. The partners of this work (three private research and/or breeding companies) have long-term experience with crossing potatoes and hops and are also involved in the cryopreservation of genetic resources of potatoes and hops in the Czech Republic. This study was initiated because there is no publication that clearly defines the thermal properties of potato and hop pollen for its cryopreservation and subsequent use for cross-breeding. The aim of this work was to determine the variability of the thermal properties of potato and hop pollen and the possibilities of its cryopreservation and subsequent use for cross-breeding. 

## 2. Results

### 2.1. Thermal Properties of Potato Pollen

**Figure 1:**
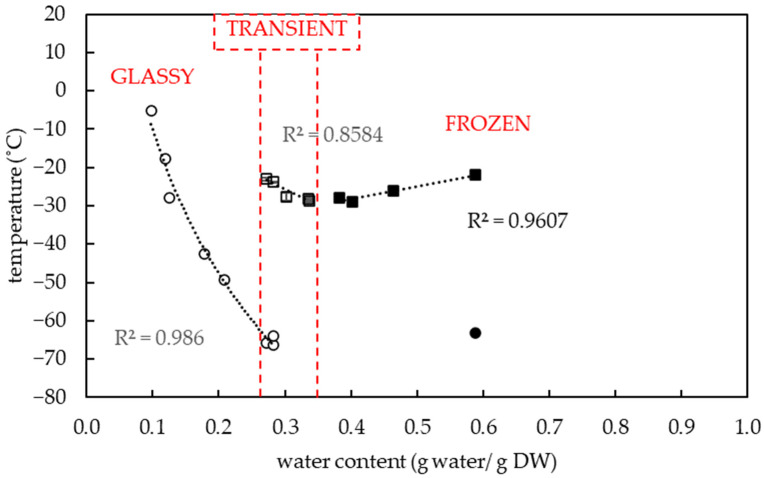
Dependence of glass transition temperature (○, ●) and melting temperature (□, ■) on total water content in potato pollen samples. The glass transition temperature was detected either with (●) or without (○) an exothermic event during sample cooling. The melting of pure water (■) and another fraction (□) showed different thermal properties. The ‘glassy’, ‘transient’ and ‘frozen’ group of pollen samples characterized by the specific thermal characteristics are indicated in the graph. Vertical bars represent the standard error of the mean from three replicates.

**Figure 2:**
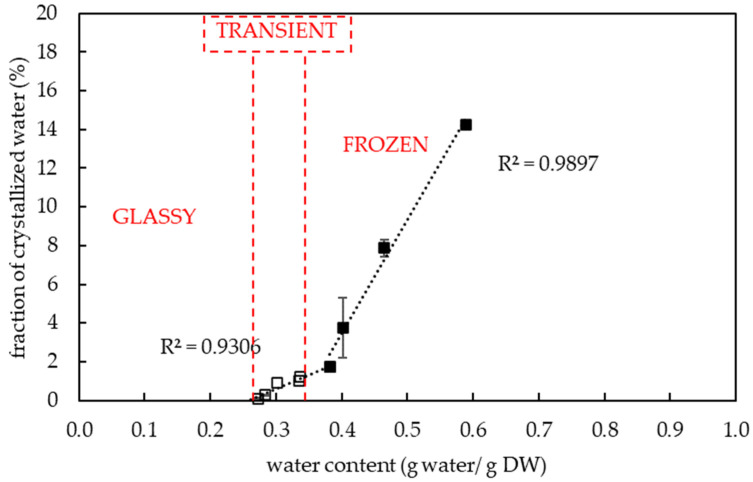
Amount of crystalline fraction expressed as a percentage of crystallized water in potato pollen depended on total water content. The melting of pure water (■) and another fraction (□) showed different thermal properties. The ‘glassy’, ‘transient’ and ‘frozen’ group of pollen samples characterized by the specific thermal characteristics are indicated in the graph. Vertical bars represent the standard error of the mean from three replicates.

Pollen samples of eight potato genotypes were collected on up to four different dates (‘Bohemia’ (3 July 2020, 13 July 2020), ‘Jasmína’ (3 July 2020, 15 July 2020), ‘Jindra’ (3 July 2020, 15 July 2020), ‘Magda’ (30 June 2020, 3 July 2020), ‘Nancy’ (3 July 2020, 15 July 2020), ‘Nautilus’ (3 July 2020), ‘Red Anna’ (15 July 2020), ‘Vlasta’ (3 July 2020, 15 July 2020), and then thermal analyses were performed on them using DSC. The results showed a correlation between the thermal characteristics of the pollen and the total water content in the evaluated samples of the selected potato varieties. (Figure 1). A total water content in potato pollen samples ranged from 0.1 to 0.59 g water per gram of DW (i.e., 9–37% WC in FW). The midpoint of the glass transition temperature of pollen samples was detected in the range from −64.3 to −5.2 °C among the genotypes tested. The amount of freezable water in pollen samples varied in a range from 0.1 to 14.3% (Figure 2).

The three specific groups of pollen samples were identified based on the presence or absence of an endothermic event and by slopes of regression lines in plots, which showed the melting temperature (Figure 1) and the fraction of frozen water (Figure 2) at specific sample water content. 

**Figure 3:**
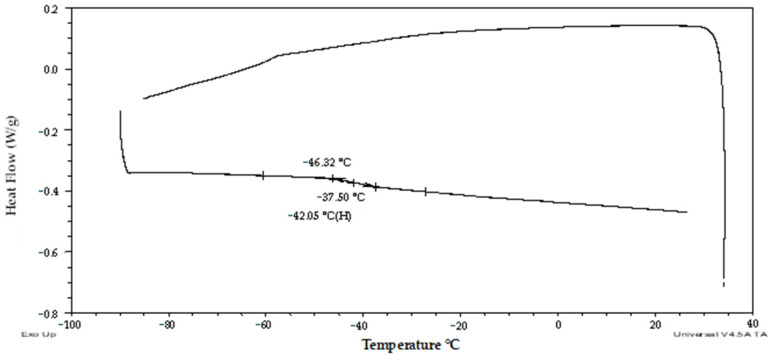
Thermogram of the ‘glassy’ sample of potato pollen with no frozen water (cv. ‘Red Anna’). The onset, endset and midpoint of the glass transition temperature is indicated.

The first group of pollen samples (’glassy’) was characterized by an absence of a significant frozen water (Figure 1, Figure 2 and Figure 3) and by the presence of glass transition (Figure 1 and Figure 3). The water content was determined in the interval from 0.1 to 0.21 g water per dry weight (Figure 1) (i.e., 9–17% WC in FW) and the glass transition temperature ranged from −49.3 to −5.2 °C. 

**Figure 4:**
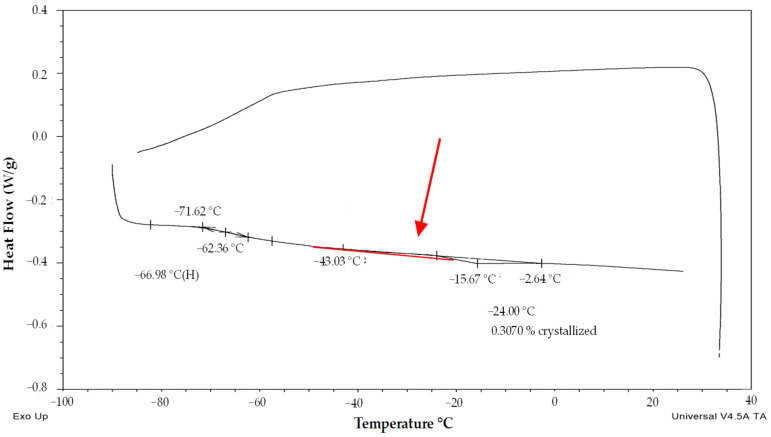
Thermogram (exo up) of the ‘transient’ sample of potato pollen with low water content (cv. ‘Nancy’). The onsets, endsets, midpoint/maximum of the glass transition/melting temperature and water crystallinity percentage are indicated. The red arrow and line show a putative exothermic event.

The second group of pollen samples (‘transient’) was specified by a negative slope of the melting curve and a slight positive slope of the frozen water fraction curve. This group of pollen samples was characterized by a moderate water content in the range from 0.27 to 0.34 g water per dry weight (Figure 1) (i.e., 21–25% WC in FW) and by very low endothermic peaks. The enthalpy of this peak corresponded to a very small proportion of frozen water (0.1 to 1.0%) (Figure 2). The melting temperature was detected in the temperature interval from −23 to −28.8 °C (Figure 1). The glass transition temperature was detected only in samples with the lowest water content in this group of samples, i.e., 0.27–0.28 g water per DW, (i.e., 21–22% WC in FW) at a temperature range from −66.4 to −64 °C (Figure 1 and Figure 4).

**Figure 5:**
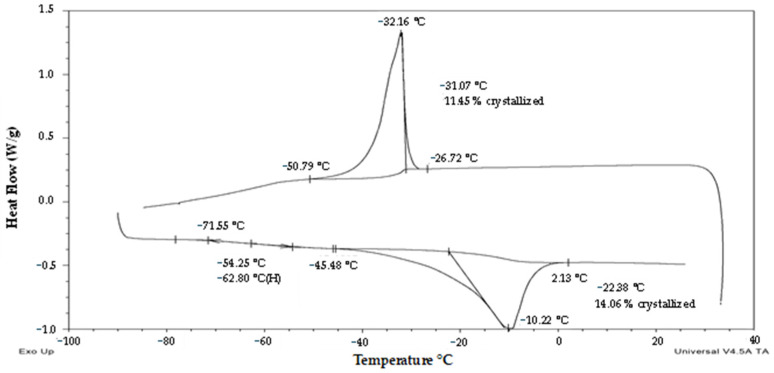
Thermogram (exo up) of the ‘frozen’ sample of potato pollen with highest water content (cv. ‘Magda’). The onsets, endsets, midpoint/maximum of the glass transition/crystallization/melting temperature and water crystallinity percentage are indicated.

**Figure 6:**
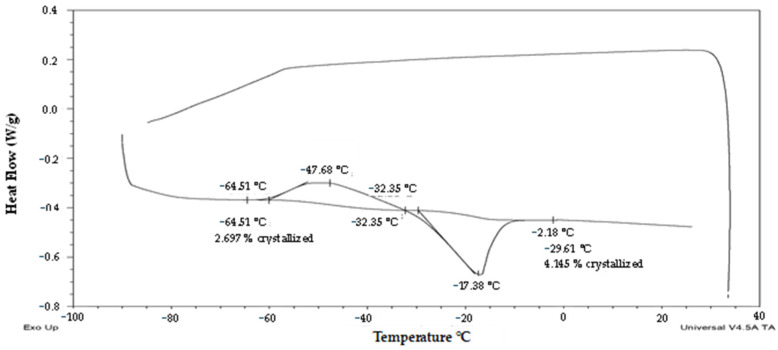
Thermogram (exo up) of the ‘frozen’ sample of potato pollen with moderate water content (cv. ‘Bohemia’). The onsets, endsets, and maximum of the crystallization/melting temperature and water crystallinity percentage are indicated.

The third group of pollen samples (‘frozen’) was characterized by a positive slope of the melting curve (Figure 1) and a steep positive slope of the frozen water fraction curve (Figure 2). A high water content in the range 0.38–0.59 g water per dry weight (i.e., 28–37% WC in FW) was related to the presence of crystallized water. The melting temperature was detected in the interval from −22 °C to −28.9 °C in this group of samples (Figure 1). The glass transition in a pollen sample was detected only in the case of the highest water content, i.e., 0.59 g water per dry weight (Figure 1), when the freezing of all water content occurred during the cooling phase of the measurement cycle (Figure 5). In other samples of the ‘frozen’ sample group, no glass transition was detected during the warming cycle, but so-called ‘cold crystallization’ or ‘devitrification’ (crystallization during warming cycle of the measurement) was always detected (Figure 6).

### 2.2. Potato Pollen Viability and Hybridization

**Figure 7:**
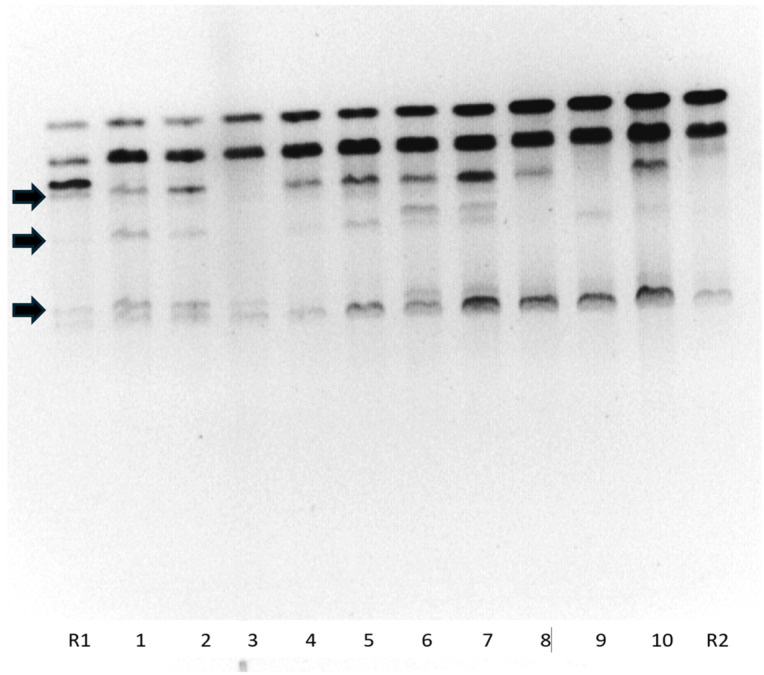
Example of electrophoretogram of DNA of seedling plants obtained using cryopreserved pollen by the RAPD method. R1—‘Gabreta’ (parental genotype—male); columns 1–10—tested outsprings (hybrids); R2—‘Larissa’ (parental genotype—female). Black arrows indicate marker fragments of the paternal genotype.

Viability of pollen samples before and after cryopreservation were evaluated with the vital staining method in fourteen selected potato varieties (Table 1). The vitality of pollen samples before cryopreservation ranged in the interval from 9 to 7 in the scale as well as the samples after cryopreservation and the tests did not reveal a significant decrease in pollen vitality after cryopreservation (Table 1) and Spearman correlation proved a similar ranking of pollen samples in both groups, before and after cryopreservation. The percentage of cross success was very low (2.3%), but productivity was comparable for potato pollen before (21 berries out of 691 crosses) and after cryopreservation (1 berry out of 44 crosses) and no statistically significant difference was found. Low hybridization success despite high pollen viability before and after cryopreservation indicates the presence of Cytoplasmic Male Sterility (CMS). In cases of successful crossing, seeds were obtained, and their hybridity was verified from seedlings (Figure 7) using DNA analysis. Some seedlings showed only the paternal fragment and the maternal fragment was missing. This was due to the heterozygosity of the (maternal) parent.

### 2.3. Thermal Properties of Hop Pollen

**Figure 8:**
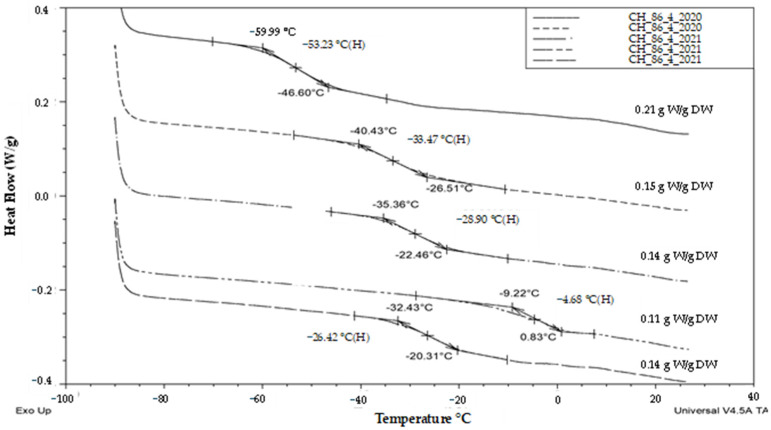
Thermograms (exo up) of hop genotype ‘86/04’ pollen taken in 2020 (two upper curves) and 2021 (three bottom curves). The onsets, endsets and midpoints of the glass transition temperatures are indicated. Water content expressed as g water (W) per g dry weight (DW) is shown on the right side of the plot.

Thermal analysis of pollen in hop male genotype ‘86/4’ collected in five different terms did not detect the presence of any significant exothermic or endothermic events that would reveal the presence of frozen water (Figure 8) in any sample tested. On the contrary, glass transition was detected in all samples. The mean glass transition value for genotype ‘86/4’ was −29.3 °C ± 6.3 (SE). In the case of hop pollen, the range of glass transition temperatures varied from −53 °C to −4.7 °C. The sample water content varied from 0.11 to 0.21 g water per g DW (i.e., 10–17% WC).

### 2.4. Hop Pollen Viability and Hybridization

**Figure 9:**
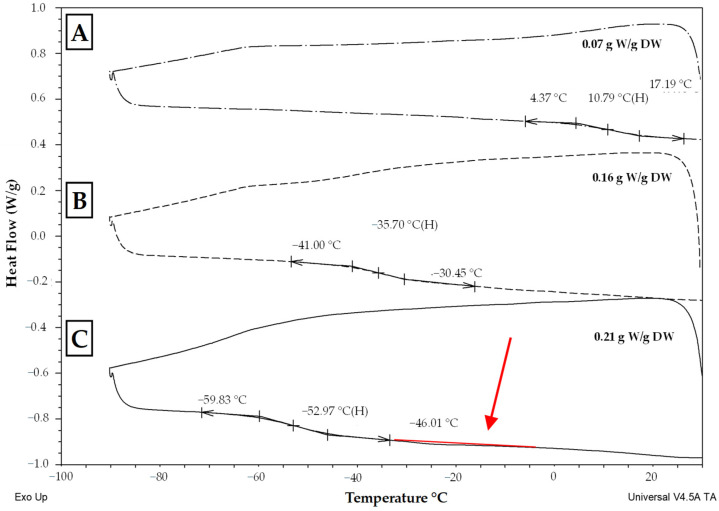
Thermograms (exo up) from thermal analysis of hop pollen genotype ‘86/4’ samples of (**A**) fresh noncryopreserved sample (86/4/2023), (**B**) sample cryopreserved for 2 years (86/4/2021) and (**C**) sample cryopreserved for 3 years (86/4/2020). The onsets, endsets and midpoints of the glass transition temperatures are indicated. Water content expressed as g water (W) per g dry weight (DW) is shown on the right side of the plot. The red arrow and line show a putative endothermic event.

**Figure 10:**
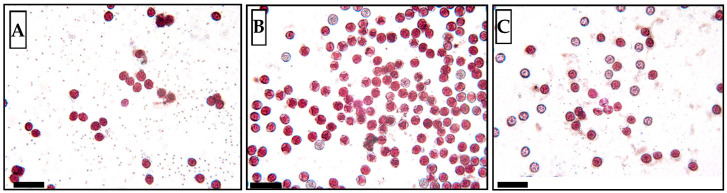
Microscopic images after vital staining with neutral red of hop pollen samples (genotype ‘86/4’) of (**A**) fresh non-cryopreserved sample (86/4/2023), (**B**) sample cryopreserved for 2 years (86/4/2021) and (**C**) sample cryopreserved for 3 years (86/4/2020). Black bars represent 50 µm, red coloured grains are fully viable pollen grains, lighter coloured grains indicate reduced viability, discoloured and deformed grains indicate pollen damage and loss of viability.

The next study compared thermal characteristics of hop pollen (genotype ‘86/4’) and its viability in a fresh sample (86/4/2023) and samples stored by cryopreservation for two (86/4/2021) or three (86/4/2020) years. When the pollen samples were evaluated using thermal analysis, all samples were found to be well dried, as no exothermic or endothermic peaks were detected (Figure 9). In contrast, glass transition was detected in all samples, but the glass transition temperature values differed significantly from each other (from −53 °C to 10.8 °C). The highest glass transition temperature (10.8 °C) was found in the freshly collected pollen (Figure 9A). The other samples were cryopreserved and analyzed after their rewarming. The thermal analysis results of these samples’ glass transition temperature in a range from −53 °C to −35.7 °C (Figure 9B,C) fitted well with the previously measured samples of hop pollen samples in a range from −53.2 °C to −4.7 °C (Figure 8). All pollen samples were treated with vital dye (neutral red) to assess pollen viability (Figure 10).

Tested pollen samples differed significantly in their viability determined by a vital staining method with neutral red dye. Freshly collected sample (Figure 10A) and the sample cryopreserved for two years (Figure 10B) showed a high pollen viability at level 9 of the ranking scale (more than 90% viability). The cryopreserved sample for three years revealed significantly lower pollen viability at level 5 (38–50%) of the ranking scale (Figure 10C) and a change of morphological characteristics in the sample was observed—the pollen was not loose but clustered. 

The hop pollen samples 86/4/2020, 86/4/2021 and 86/4/2023 were removed from liquid nitrogen and experimental pollination of female hop plants was performed in years 2022 and 2023 at the Hop Research Institute with selected mother hop plants (genotype ‘Eris’). Both fresh and cryopreserved hop pollen samples showed high fertility, good pollination ability and subsequent seed production (Table 2) with the sole exception of sample 86/4/2020 in hybridization year 2023, when this sample did not show seed production. This pollen sample was used for pollination only in one pollination term (31 July 2023) in 2023, and its vitality test revealed lower vitality by the vital staining method (Figure 10C). On the other hand, one pollination term (9 August 2022) with the same sample was sufficient to pollinate in 2022. The obtained seeds from all crossings showed no signs of abnormalities and were sown for growing hybrid seedlings and their subsequent testing in the hop breeding process.

## 3. Discussion

Thermal analysis and viability tests of potato and hop pollen brought new knowledge that can be used for their cryopreservation and utilization in the breeding process. The analysis of fresh potato pollen (Figure 3, Figure 4, Figure 5 and Figure 6) showed high variability in thermal characteristics and viability of the tested samples, while for the model male hop genotype (Figure 8 and Figure 9) no significant presence of first-order phase transitions was observed in any of the tested samples. On the other hand, no significant differences were observed in potato pollen viability in samples before and after cryopreservation (Table 1). Thus, the natural variability of samples before cryopreservation had a higher effect on the pollen fertility than their storage in liquid nitrogen. Thermal analyses results of pollen samples helped to explain the differences in pollen properties and their viability. 

The thermal analysis of potato pollen samples allowed one to distinguish three pollen groups differing in their thermal characteristics and crystalline fraction values (Figure 1 and Figure 2). These groups were defined by the presence or absence of the first- or second-order transitions of matter in samples and by a slope of regression lines in plots that showed the melting temperature (Figure 1) and the crystalline fraction (Figure 2) at specific sample water content. We briefly classified these three groups of pollen samples based on different thermal characteristics and water content as (1) ‘glassy’, (2) ‘transient’, (3) ‘frozen’. Three other groups of pollen samples were identified by DSC earlier by a slope of curves of the relationship Cp–water content [28] in the interval of 0.01 to 0.3 g water per DW in *Typha latifolia* L. and *Zea mays* L. Despite the different measured parameters and the ranges of water content, some findings are comparable and are discussed below. 

The first group of pollen samples (‘glassy’) in our study did not show any exothermic/endothermic event and was always characterized by the glass transition presence and by a very low water content in the interval from 0.1 to 0.21 g water per dry weight or 9–17% WC (Figure 1, Figure 2 and Figure 3). Similarly, water freezing in pollen samples was not detected by a DSC method at the same cooling/warming rates in wheat pollen samples with water content lower than 0.28 g water per DW [40]. Absence of water freezing was also observed at water content values less than 0.21 and 0.26 g per DW in *Typha latifolia* L. and *Zea mays* L., respectively [28]. Liquid water turned into a solid state amorphous glass without ice formation in the first group of pollen samples in our study. The glass transition temperature increased when water content decreased similarly as other authors presented [30]. The ‘glassy’ pollen was also identified among all hop pollen samples in our study (Figure 8 and Figure 9). No hop pollen sample showed obvious water crystallization and the water content ranged in a low level from 0.07 to 0.21 g water per g DW. Since the hop pollen samples were always well dehydrated (unlike the potato samples), we hypothesize that hop pollen naturally tends to be properly dehydrated, as pollen longevity in dry, adverse conditions is more important in pollination of anemophilic and dioecious plant species as hop compared to entomophilic and self-fertilizated species such as potato. This could also explain the high variability of the thermal characteristics and viability of potato pollen, which are apparently more influenced than hops by the conditions of collection, transfer and short-term storage of freshly collected pollen.

The second (‘transient’) group of pollen samples, at medium water content, showed small, negligible, endothermic events (Figure 1, Figure 2 and Figure 4). These samples were characterized by a medium water content (0.27 to 0.34 g water per dry weight or 21–25% WC) (Figure 1) and very small events of the first order transitions in potato pollen. The glass transition in this group of pollen samples was difficult to identify by a standard DSC method and was detected only in samples with a water content of 0.27–0.28 g water per DW, when the glass transition was not masked by an exothermic peak. Exothermic events occurred always during the warming part of the measurement cycles. Endotherm onset temperatures did not decrease with decreases in the total water content as in the third group of pollen samples occurred (Figure 1). However, the amount of enthalpy decreased with decreasing water content, but at a different slope, when compared with the third group of pollen samples (Figure 2). The enthalpy of these endothermic events was expressed as a percentage of frozen water; therefore, ice crystals were assumed to be present. This corresponded to a very low frozen water content (0.1 to 1.0%). Understanding and explaining this endothermic event is not simple. The increase of onsets of these endothermic peaks with decreasing water content showed that this event cannot represent a melting of pure ice crystals. Some authors consider this endothermic event as the melting of neutral lipids because the size of the peak and transition temperature seemed to be independent of the WC [28]. On the other hand, the authors stressed the impact of other substances in pollen cryopreservation as sugars, proteins and some other components [8]. We consider that the decrease in size of this endothermic event with decreasing water content in our study indicates some role of water. We suggested this event as a result of crystallization of some complex system including water and some solute(s)—sugars or other hydrophilic substances. A similar small event was identified as a result of solute (sucrose) inclusions [41] in highly concentrated sucrose solutions close to the concentration of the so-called maximally freeze-concentrated phase [34]. So, we can accept two hypotheses: the small endothermic events in dehydrated systems may represent either (1) solute inclusions or (2) complex system containing both solute and water crystals. However, this negligible endothermic event can potentially have a negative effect on pollen viability. This claim can be at least partially supported by our results of hop pollen viability and fertility tests. A hop pollen sample showed reduced viability in year 2023 after three years of its cryopreservation (Figure 10C) and the failure of the hybridization process (Table 2). The interpretation of this result is not entirely clear because the same pollen sample was successfully used for hybridization in year 2022, and because pollination did not follow the standard procedure as only one pollination term was used in year 2023 instead of the standard use of two terms in year 2022. Repeated detailed analysis of this sample allowed one to identify a slight deflection of the heat-flow signal line (Figure 9), which could be interpreted as a very small endothermic peak, which is not significant and is at the limit of detectability of the method and equipment used. This theory could be supported by the low value of the glass transition temperature (−53 °C) correlated with a relatively high water content (0.21 g water per g DW) detected in the pollen sample. We conclude that the negligible endothermic event in the ‘transient’ group of pollen samples may indicate reasons for the reduced viability of the pollen sample and the reduced reliability of hybridization with the sample. 

The third group of pollen samples (‘frozen’), at the highest water content, showed significant exothermic/endothermic peaks (Figure 1, Figure 2, Figure 5 and Figure 6) as a result of a high water content (>0.38 g water per g DW or >28% WC). Only at the highest water content value of 0.59 g water per g DW (38% WC) did all water crystallize during the cooling cycle at a cooling rate of 10 °C per minute. In other samples of the ‘frozen’ pollen category, a part of water crystallized during warming cycles (so-called ‘cold crystallization’ or ‘devitrification’). This effect was observed in the similar region of 0.25–0.35 g water per DW in the former study [28]. Thanks to this effect, the glass transition was identified only in the sample with the highest water content in our study; as in the other samples, the glass transitions were masked by the cold crystallization. The results proved that the area and temperature of the melting peak increased with increasing WC [28].

When the results of this study are applied, it should be taken into account that the results of the thermal analysis correspond to standard rates of cooling and heating of 10 °C per minute. In the case of higher cooling and heating rates, it would be possible to overcome the risk of freezing in samples with a higher water content.

## 4. Materials and Methods

### 4.1. Pollen Sample Collections

Pollen samples were taken from plants at the workplaces of VESA Velhartice (Velhartice, Czech Republic) and the Potato Research Institute Havlickuv Brod, Ltd. (PRI) (Havlickuv Brod, Czech Republic), or Hop Research Institute Co., Ltd. (HRI) (Zatec, Czech Republic). Potato varieties with different flowering times and different pollen yields (in the indicated years) were selected: ‘Bohemia’ (2022), ‘Doubrava’ (2022), ‘Gabreta’ (2022), ‘Granola’ (2019), ‘Jasmína’ (2022), ‘Jindra’ (2019, 2020, 2022), ‘Magda’ (2020, 2022), ‘Nancy’ (2020, 2022), ‘Nautilus’ (2020), ‘Queen Anne’ (2022), ‘Red Anna’ (2020), ‘Saprodi’ (2022), ‘Stilleto’ (2019), ‘Troja’ (2019), ‘Valfi’ (2019), ‘Vlasta’ (2020). At the Hop Research Institute, male genotype of hops ‘86/4’ was selected for testing the thermal properties and viability of the pollen in years 2020, 2021, 2022 and 2023.

**Figure 11:**
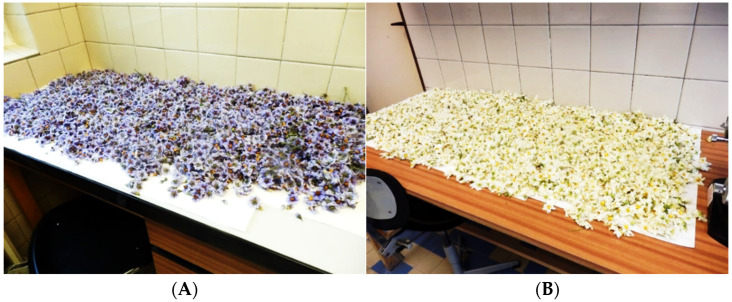
Freshly picked flowers of the potato varieties ‘Valfi’ (**A**), ‘Jasmína’ (**B**).

**Figure 12:**
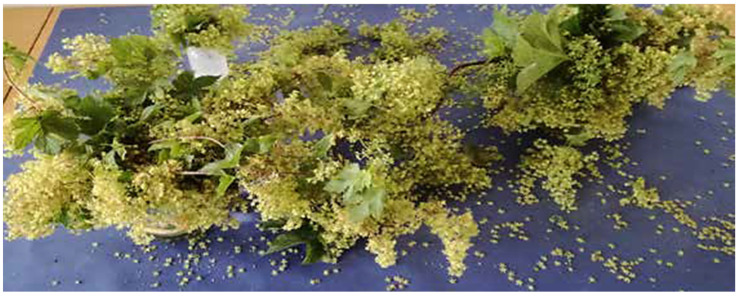
The axils of the male hop plant.

Standard procedures for collection of potato and hop pollen during standard cross-breeding in breeding companies were used. After the anthers matured, potato inflorescences were removed from the mother plants and placed in laboratory conditions (Figure 11) for 1 day. After that, pollen was released by tapping the individual anthers with a needle. The pollen was then poured into plastic tubes and stored in the cold (4 °C). In the case of hops, male inflorescences were taken from selected genotypes of male hop plants. In the course of 3 days in laboratory conditions, the inflorescence dried up and spontaneously released pollen grains (Figure 12). The pollen was then poured into plastic tubes and stored in the cold (4 °C).

### 4.2. Thermal Analysis

The method of differential scanning calorimetry was used to determine the temperature of phase transitions and the value of the melting enthalpy in pollen samples of selected potato varieties, ‘Bohemia’, ‘Jasmína’, ‘Jindra’, ‘Magda’, ‘Nancy’, ‘Nautilus’, ‘Red Anna’, ‘Vlasta’, at up to four different collection days. The male genotype ‘86/4’ was used to test thermal properties in hops. A differential scanning calorimeter Q2000 with RCS or Discovery Multi-Sample X3 DSC with RCS (TA Instruments—Waters LLC, New Castle, DE, USA) was used in the range −90 to +30 °C, at a cooling and heating rate of 10 °C/min, using hermetically sealed aluminium pans. During heating, the glass transition temperature, the melting temperature expressed as the peak onset, the amount of heat of fusion and the amount of frozen water in the sample were calculated and expressed as a percentage of the total weight of the sample. The total water content in the sample was determined gravimetrically from the difference between fresh weight and dry weight after drying the sample to a constant weight in a hot air dryer at a temperature of 105 °C.

### 4.3. Pollen Viability Evaluation

Viability of pollen samples before and after cryopreservation were evaluated in selected potato varieties and specified years ‘Bohemia’ (2022), ‘Doubrava’ (2022), ‘Gabreta’ (2022), ‘Granola’ (2019), ‘Jasmína’ (2022), ‘Jindra’ (2019, 2022), ‘Magda’ (2022), ‘Nancy’ (2022), ‘Queen Anne’ (2022), ‘Saprodi’ (2022), ‘Stilleto’ (2019), ‘Troja’ (2019), ‘Valfi’ (2019) using the iodine staining method [42]. Pollen grains were stained with a solution of 1% iodine and potassium iodide with a trace amount of acid fuchsin. To prepare the staining solution, 1 g of iodine was first dissolved in a sufficient amount of 96% ethanol; this solution was diluted with the addition of distilled water to a volume of 100 mL. A trace amount of acid fuchsin was added to this solution. Next, 1 g of potassium iodide was dissolved in 100 mL of distilled water. The solutions were stored in the dark until use. Both solutions were mixed together in a 1:1 ratio before being used to stain pollen grains. The day before the determination of fertility, the flowers were plucked. The next day, the pollen from the flowers was knocked onto the hourglass. Slides were viewed under a Jenalumar microscope at 500× magnification. Potato pollen viability was assessed by the shape and intensity of the coloration of the pollen grains.

Fresh hop pollen samples were stained using modified vital staining procedures [43] using 0.3% neutral red (Roth) vital stain solution. Pollen from the flowers was tapped onto a microscope slide, a drop of neutral red staining solution was added, and a microscope coverslip was attached. Treatment with the staining solution was extended for 30 min, after which the staining solution was washed out with 0.9% sodium chloride solution. Microscopic images were taken using the Carl Zeiss Amplival microscope using 40× objective, digital CMOS 570 camera and Capture software, version 2.1 and the proportion of fully viable pollen grains was determined from the captured images taken. The viability of hop pollen was assessed according to the shape and intensity of the coloration of the pollen grains, just as with potatoes. Five microscope slides were prepared from each pollen sample to determine its fertility. On each slide, the number of pollen grains was evaluated in four microscopic fields of view at different locations of the preparation. The number of rounds, coloured pollen grains (fertile) and uncoloured, wrinkled, deformed and black-brown coloured grains was expressed as a percentage, and an arithmetic mean expressing the average pollen fertility on one preparation was calculated. The average fertility of the five preparations was then calculated from these averages. Subsequently, the average fertility in percentages was converted into a nine-point viability rating scale developed for a routine pollen viability assessment at the Potato Research Institute Havlíčkův Brod (Table 3).

### 4.4. Potato Pollination

**Figure 13:**
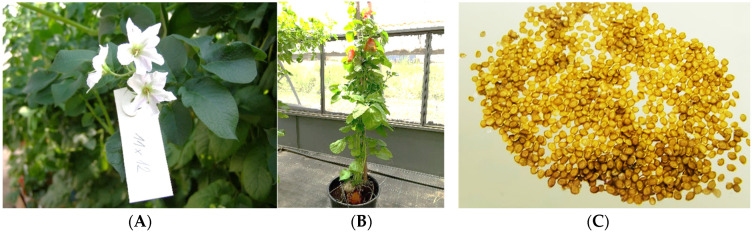
Removing anthers from potato flowers to prevent self-pollination (**A**). A potato berry in a nylon knit bag (**B**). Potato seeds resulting from crossing prepared for sowing seedlings (**C**).

After cryopreservation, the pollen of tested varieties was transferred from the Crop Research Institute to VESA Velhartice for the purpose of pollination (Figure 13). A total of 735 cross combinations were crossed with the transferred pollen. Of the total number, 691 were crossed with fresh potato pollen and 44 with pollen after cryopreservation. Seeds obtained from crossing with pollen after cryopreservation in 2022 (Larissa × Gabreta) were sown. The hybridity of these seedlings was verified using DNA analysis. DNA was extracted from aerial parts of plants using the commercial GeneElute Plant Genomic DNA Isolation Kit (Sigma). The isolated DNA was also used to confirm or refute the hybridity of individual hybrids by the RAPD method. The amplification reaction was carried out in 20 mL volumes and contained 200 ng template DNA, 10 mL FastStart PCR Master (Roche), 3.2 mM primer (IDT) and water (primer P72: CGGCCACTGT was used). The RAPD reaction took place in the XP cycler thermocycler (Bioer), where the following course of the reaction was programmed: initial 3 min of denaturation at 94 °C; followed by 40 cycles of 1 min at 94 °C, 1 min 40 s at 37.5 °C, and 2 min at 72 °C; the final step was 10 min at 72 °C followed by cooling to 4 °C. Electrophoresis was performed in a 1.5% agarose gel with ethidium bromide. 

### 4.5. Hop Pollen Longevity Test and Pollination

**Figure 14:**
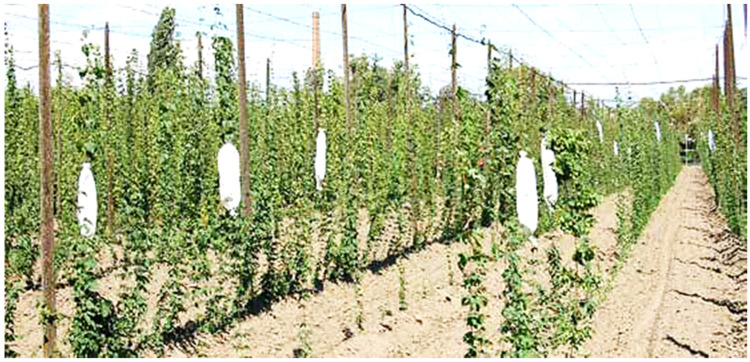
Isolation of inflorescences of female hop plants used for crossing.

Four different pollen samples of male genotype ‘86/4’ were used for the tests: (1) fresh pollen from the 2023 harvest year, (2) pollen harvested and cryopreserved in the year 2023, (3) pollen harvested and cryopreserved in the year 2021, (4) pollen harvested and cryopreserved in the year 2020. After two or three years of cryopreservation, pollen samples were thawed and thermal and vitality analyses were performed. The pollination of maternal plants of cv. ‘Eris’ (Figure 14) was performed with the fresh pollen samples and with <1-, 2- and 3-year cryopreserved pollen samples in years 2022 and 2023 with the male genotype ‘86/4’. The seed development and TSW (thousand seed weight) was determined after harvest, but the number of seeds was not evaluated because the control (non-cryopreserved) samples were treated with a larger volume of pollen (min 1 mL) compared to the cryopreserved hop pollen samples (200 μL). 

### 4.6. Statistical Analyses

Statistically significant differences were measured using Statistica software vers. 14.0.0.15 (TIBCO Software Inc., Palo Alto, CA, USA). The following tests were used to assess the differences: Student’s *t*-test to evaluate the averages of potato pollen viability before and after cryopreservation, Spearman’s correlation coefficient to assess the rank correlation of potato pollen viability of individual varieties before and after cryopreservation and test of differences between two proportions to compare the success of potato pollen crossing before and after cryopreservation or compare the proportion of viability in a hop longevity test.

## 5. Conclusions

This study provides new and applicable results in the field of thermal characteristics of potato and hop pollen and complements previous findings from the thermal analysis of pollen from other plant species. Thermal analysis of potato pollen identified three specific groups of samples differing in total water content and the presence of a first- and second-order mass transition. The low water content of the potato and hop pollen samples (‘glassy’) (<0.21 g water per dry matter) excluded freezing of water during pollen cryopreservation and the presence of a glassy state was detected. Cryopreservation of this properly dehydrated pollen did not lead to its damage. The high water content of pollen (‘frozen’) samples (>0.34 g water per g DW) always resulted in the presence of first-order transition events—exothermic and endothermic peaks indicating a high risk of pollen damage during cryopreservation. The ‘transitional’ pollen samples were characterized by moderate water content (0.27–0.34 and 0.21 g water per g DW in potato and hops, respectively) and the presence of small endothermic events, melting of either solute inclusions or crystals of some complex system could reduce the viability of the pollen and the efficiency of the hybridization process. Hop pollen generally showed better thermal properties for its cryopreservation compared to potato and never showed significant frozen water content in the samples. Potato pollen samples showed large natural variation in total water content and its frozen fraction, as well as variation in pollen viability. This finding indicates the high sensitivity of potato pollen to collection conditions and short-term storage of samples compared to hop pollen. In contrast to hop pollen, potato pollen showed high variability in thermal properties and water content; therefore, more attention should be paid to the preparation of potato pollen and moisture control before its cryopreservation. The near-zero glass transition temperature detected in some pollen samples by thermal analysis can help to set a safe storage temperature and carry out their cryopreservation in conventional freezers without the need for liquid nitrogen technologies. 

The study contributes to the knowledge and practical use of cryopreservation and the use of plant genetic resources. Using differential scanning calorimetry, a simple and effective method of preserving potato and hop pollen for breeding purposes was verified. DSC has proven to be a suitable tool for measuring and controlling pollen endotherms and glass transition temperatures. The study thus enables a wider use of the cryopreservation method for plant breeding and hybridization, as it enables the storage and transport of pollen from different sources and locations and overcomes the limitations preventing the crossing of plants that have different flowering times, dioeciousness or current lack of pollen availability.

## Figures and Tables

**Table 1:** Viability of potato pollen of selected varieties before and after cryopreservation.

Genotype	Year of Cryopreservation	Viability Rating Scale	
		Before CRYO	After CRYO
Jindra	2022	9	9
Bohemia	2022	8	9
Saprodi	2022	8	9
Jindra	2019	8	8
Granola	2019	8	7
Valfi	2019	8	7
Doubrava	2022	8	7
Gabreta	2022	8	7
Queen Anne	2022	8	7
Troja	2019	7	8
Jasmína	2022	7	8
Stilleto	2019	7	7
Magda	2022	7	7
Nancy	2022	7	7
Mean		7.7 ± 0.17 SE	7.6 ± 0.23 SE

**Table 2:** Hop pollen hybridization results with the mother’s genotype cv. ‘Eris’ and the father’s genotype ‘86/4’. TSW represents the thousand seed weight of sample.

Variant	Year of		Pollination		Seed	
	Cryopreservation	Pollination	First	Second	Production Success	TSW (g)
FRESH	none	2023	31-Jul	03-Aug	yes	1.8
CRYO	2023					3.17
	2021					1.36
	2020			none	no	-
FRESH	none	2022	09-Aug	12-Aug	yes	3.3
CRYO	2022					3.16
	2021					3.33
	2020			none		4.29

**Table 3:** Standard pollen viability ranking scale.

Pollen Viability		
Ranking Scale	Assessment	(%)
1	very low	0
2		0.1–12.5
3	low	12.6–25.0
4		25.1–37.5
5	medium	37.6–50.0
6		50.1–62.5
7	high	62.5–75.0
8		75.1–87.5
9	very high	87.6–100

## Data Availability

The data that support the findings of this study are available from the corresponding author, M.F., upon reasonable request.
